# Use of medicines by adults in Germany

**DOI:** 10.17886/RKI-GBE-2017-130

**Published:** 2017-12-13

**Authors:** Hildtraud Knopf, Franziska Prütz, Yong Du

**Affiliations:** Robert Koch Institute, Department of Epidemiology and Health Monitoring, Berlin

**Keywords:** MEDICINES, SELF-MEDICATION, ADULTS, HEALTH MONITORING, GERMANY

## Abstract

The use of medicines is an essential aspect of treating disease. In this field, surveys that map the population’s use of medicines are of particular interest. The GEDA 2014/2015-EHIS study collected data on the use of medically prescribed and self-medicated drugs during the two weeks that preceded the survey. 58.9% of women and 52.0% of men reported that they had taken medically prescribed drugs during this period. 48.5% of women and 35.4% of men stated they had taken medication during this period that had not been prescribed by a doctor. The prevalence of the use of medically prescribed drugs and self-medication was higher among women than among men. Finally, the prevalence of the use of medically prescribed drugs increased significantly with age, whereas the prevalence of self-medication decreased with age.

## Introduction

Medication is an essential to the treatment of health impairments, disorders and diseases. Alongside expenses for outpatient and inpatient services, expenditure on medicine is one of the major costs covered by statutory health insurers (SHIs). In 2016, German SHIs paid out EUR 38.5 billion for drug therapy [1]. These figures are supplemented by the costs of medicines borne by the patients themselves through self-medication which amounted to around EUR 6.4 billion in 2015 [2]. A valid analysis of the consumption of medicines (the number of medicines sold) and use (the amount of medicine that is actually taken) is not only important from an economic point of view; it is a vital to assessments of the population’s health and to quantifying the use of medical services.

Since not all medically prescribed drugs are actually used [3] and only medically prescribed drugs are covered by SHI statistics [4], surveys that map use, including self-medication by the population are of particular importance. Data from the health surveys conducted by the Robert Koch Institute (RKI) as part of the health monitoring framework can bridge the gap in the SHI data. The European Health Interview Survey (EHIS) provides harmonised health data that can be compared with those from other European Union member states (the EU, as well as Norway and Iceland). The EHIS questions on the use of medically prescribed drugs and over-the-counter, pharmacy-only medicines as well as those sold in chemists and supermarkets as over-the-counter preparations were integrated into the German Health Update study (GEDA 2014/2015) conducted by the RKI. The GEDA 2014/2015-EHIS study, therefore, provides up-to-date data on the use of medicines in Germany.

**GEDA 2014/2015-EHIS**

**Data holder:** Robert Koch Institute

**Aims:** To provide reliable information about the population’s health status, health-related behaviour and health care in Germany, with the possibility of a European comparison

**Method:** Questionnaires completed on paper or online

**Population:** People aged 18 years and above with permanent residency in Germany

**Sampling:** Registry office sample; randomly selected individuals from 301 communities in Germany were invited to participate

**Participants:** 24,016 people (13,144 women; 10,872 men)

**Response rate:** 26.9%

**Study period:** November 2014 - July 2015

**Data protection:** This study was undertaken in strict accordance with the data protection regulations set out in the German Federal Data Protection Act and was approved by the German Federal Commissioner for Data Protection and Freedom of Information. Participation in the study was voluntary. The participants were fully informed about the study’s aims and content, and about data protection. All participants provided written informed consent.

More information in German is available at www.geda-studie.de

## Indicators

The prevalence of current use is represented by the figures on the use of medication that occurred in the two weeks prior to the survey. The data was divided between two indicators as a distinction was made between the use of medically prescribed drugs and over-the-counter medicines. Data on these two indicators were collected as part of the GEDA 2014/2015-EHIS study using a self-administered questionnaire completed on paper or online. The questionnaire asked: 1) ‘Have you taken any medications prescribed by a doctor in the last 2 weeks? (Do not include the pill or other hormonal contraceptive preparations.)’ and 2) ‘Have you taken any medications, herbal remedies or vitamins that were not prescribed by a doctor during the last 2 weeks? (Do not include the pill or other hormonal contraceptive preparations.)’

The analyses of prescribed drugs are based on data from 23,898 participants aged 18 or over (13,087 women; 10,811 men) with valid data on the use of medicines. The analyses of self-medication are based on information from 23,848 individuals (13,063 women; 10,785 men). The calculations were carried out using a weighting factor that corrected the sample for deviations from the population structure (as of 31 December 2014) in terms of gender, age, district type and level of education. The district type reflects the degree of urbanisation and corresponds to the regional distribution in Germany. The International Standard Classification of Education (ISCED) was used to classify the educational and occupational qualifications [5]. A detailed description of the methodology used for GEDA 2014/2015-EHIS can be found in Lange et al. 2017 [6] as well as in the article German Health Update: New data for Germany and Europe in issue 1/2017 of the Journal of Health Monitoring. This study describes prevalences with 95% confidence intervals (95% CI). A statistically significant difference between groups is assumed when p-values are lower than 0.05 (after taking weighting and the survey design into account). The results presented here are purely descriptive and do not enable conclusions to be drawn as to whether other factors may explain the differences that were identified between the groups.

## Results and discussion

More than half (55.5%) of all study participants (58.9% of women and 52.0% of men) reported that they had taken medically prescribed drugs during the two weeks prior to the study (Table 1).

Although the observation period for GEDA 2014/2015-EHIS was longer than the period analysed for the 2008-2011 German Health Interview and Examination Survey for Adults (DEGS1) – two weeks instead of one week – GEDA 2014/2015-EHIS identified a significantly lower prevalence among women than the rate found by DEGS1 (58.9% instead of 71.3 %) [6]. However, GEDA 2014/2015-EHIS identified a higher prevalence among men (52.0% instead of 46.1%) [6]. The differences among women can probably be explained by the fact that GEDA 2014/2015-EHIS did not record the use of the pill or other contraceptive hormonal preparations. The higher rate identified among men is probably due to the longer observation window used in GEDA 2014/2015-EHIS. Most importantly, however, DEGS1 and GEDA 2014/2015-EHIS studied different age groups (18 to 79 and 18 or above) and also differed on the way in which they implemented data collection.

GEDA 2014/2015-EHIS demonstrates that the use of medically prescribed drugs increases with age: in the youngest age group (18 to 29), 33.8% of women and 21.3% of men reported that they had taken medicines during the past two weeks, whereas prevalence rates for those aged 65 or above were much higher (87.1% among women and 86.3% among men, Table 1). This increase has also been demonstrated by other studies [7] and is attributable to the fact that the prevalence of (chronic) diseases rises with age. GEDA 2014/2015-EHIS did not record the actual number of medicines that were taken. However, DEGS1 demonstrates that over 40% of 70- to 79-year-old adults took at least five medically prescribed drugs in the seven days prior to the survey [7]. In the light of the potential risks of polypharmacy [8-11], this result demonstrates the need for further research. A direct comparison of the age-specific prevalences identified by GEDA 2014/2015-EHIS and DEGS1 cannot be undertaken, as the studies targeted different age groups [7].

Significant differences in the use of medically prescribed drugs between the genders in the 18-to-29, 30-to-44, and 45-to-64 age groups were identified from the data collected for GEDA 2014/2015-EHIS. The prevalence was higher among women than men, but converged among people aged above 44. DEGS1 also observed that gender-specific differences tend to smooth out in older age [7].

Apart from people aged 65 or above, GEDA 2014/2015-EHIS found that women and men from the lower educational group had a higher prevalence of using medically prescribed drugs than those from the upper educational group. The differences were significant in women aged 18 to 29 and 45 to 64, as well as in men aged 30 to 44 and 45 to 64 (Table 1). DEGS1 also identified a higher use of medicines among people with a low socioeconomic status, and in particular among women [7].

Almost half (48.5%) of women and more than one-third (35.4%) of men reported that they had taken medications that had not been medically prescribed, in other words, that they had self-medicated during the two weeks that preceded the GEDA study (Table 2). As with medically prescribed drugs, the prevalence of self-medication among women was significantly higher than among men. This difference can be observed in all age groups. In contrast to the age-specific increase in the use of medically prescribed drugs, self-medication tends to decrease with age. Self-medication was much less common in people aged 45 or above, than among under-45s. In terms of educational status, prevalence rates were higher for the upper educational group than for the lower educational group. However, differences are significant only among women aged 30 or over and among men aged 45 to 64 (Table 2). The increase in self-medication with rising educational attainment, which is often associated with a higher professional status and higher income, has also been previously demonstrated by DEGS1 and the German National Health Interview and Examination Survey 1998 (GNHIES98, 1997-1999). These studies show that self-medication was associated with high levels of social status among both genders [7, 12, 13]. Similar results were obtained from a population-based study in Spain, where self-medication in the case of acute diseases was also associated with higher levels of education [14].

There are only slight differences between the federal states in terms of the use of medicines (Data not shown). Compared to the national average, significantly lower prevalences of the use of medically prescribed drugs are found among women in Hamburg, Bavaria and Baden-Württemberg, as well as among men in Bremen and Berlin. Higher prevalences were identified among women and men in Saarland, among women in Brandenburg and among men in Saxony-Anhalt. Significantly lower prevalences of self-medication were observed in Thuringia and Saxony-Anhalt among both genders, among women in Mecklenburg-Western Pomerania and among men in Baden-Württemberg. Higher prevalences were only observed among women in Bavaria, Rhineland-Palatinate, and Baden-Württemberg. Data on the regional distribution of the use of medicines according to federal state are available from the Information System of the Federal Health Monitoring (www.gbe-bund.de). With regard to prescription medicines, demographic factors such as age, gender, the social situation and the morbidity spectrum of each federal state are important, but not the only reasons that explain regional differences [15-17]. Differentiated data on the self-medication of non-medically prescribed drugs are, as yet, only available from GEDA 2014/2015-EHIS. The analysis of the differences between the old German federal states and the new federal states undertaken as part of GNHIES98 showed a much lower level of self-medication among people living in the new federal states [13].

Medicines are essential to health care. This Fact sheet, along with other contributions to this issue (Fact sheets on the utilization of outpatient and inpatient medical care, physiotherapy, and the Focus on the utilization of psychotherapeutic and psychiatric treatment), provides an overview of significant aspects of the utilization of health care by adults in Germany. Continuous monitoring of the consumption of medicines is of particular relevance to public health with regard to both cost and health issues. However, it is not only medically prescribed drugs that need to be considered; studies also need to cover everything that is used for self-medication. The importance of self-medication is underscored by an international comparison that places Germany as one of the table leaders [18].

## Key statements

- More than half of the study participants took medication that had been prescribed by a doctor during the two weeks prior to the study.
- Almost half of the women and more than a third of the men surveyed took medication that had not been prescribed by a doctor within the two weeks prior to the study.
- Although the prevalence of the use of medically prescribed drugs increases significantly with age, the prevalence of self-medication tends to decrease with age.
- With the exception of people aged 65 or above, the prevalence of the use of medically prescribed drugs is higher among women than in men; the prevalence of self-medication is higher among women of all ages.

## Figures and Tables

**Table 1:** Prevalence of the use of medically prescribed medicines in the last 2 weeks according to gender, age and educational level (n=13,087 women, n=10,811 men) Source: GEDA 2014/2015-EHIS

Women	%	(95% CI)	Men	%	(95% CI)
**Women (total)**	**58.9**	**(57.8-60.0)**	**Men (total)**	**52.0**	**(50.8-53.2)**
**18-29 years**	33.8	(31.4-36.4)	**18-29 years**	21.3	(18.8-24.1)
Low education	40.5	(34.3-46.9)	Low education	25.6	(20.1-32.1)
Medium education	33.1	(29.9-36.5)	Medium education	20.1	(17.1-23.5)
High education	27.5	(23.3-32.2)	High education	19.0	(14.2-24.9)
**30-44 years**	38.7	(36.6-40.8)	**30-44 years**	31.9	(29.4-34.4)
Low education	41.7	(34.9-48.9)	Low education	36.9	(29.2-45.4)
Medium education	39.3	(36.5-42.1)	Medium education	35.3	(32.1-38.7)
High education	35.4	(32.1-38.8)	High education	22.9	(20.2-25.9)
**45-64 years**	61.4	(59.8-63.1)	**45-64 years**	58.5	(56.6-60.4)
Low education	67.0	(62.5-71.3)	Low education	67.0	(62.2-71.5)
Medium education	61.5	(59.4-63.5)	Medium education	59.7	(56.9-62.5)
High education	56.2	(53.4-58.8)	High education	53.5	(50.9-56.0)
**≥ 65 years**	87.1	(85.5-88.5)	**≥ 65 years**	86.3	(84.9-87.7)
Low education	88.7	(86.5-90.7)	Low education	86.3	(82.6-89.3)
Medium education	85.9	(83.4-88.0)	Medium education	87.0	(84.7-89.1)
High education	85.9	(82.7-88.7)	High education	85.2	(82.7-87.4)
**Total (women and men)**	**55.5**	**(54.7-56.4)**	**Total (women and men)**	**55.5**	**(54.7-56.4)**

CI=confidence interval

**Table 2:** Prevalence of the use of non-medically prescribed medicines (self-medication) over the past 2 weeks according to gender, age and educational level (n=13,063 women, n=10,785 men) Source: GEDA 2014/2015-EHIS

Women	%	(95% Cl)	Men	%	(95% Cl)
**Women total**	**48.5**	**(47.3-49.6)**	**Men total**	**35.4**	**(34.2-36.6)**
**18-29 years**	51.3	(48.7-53.8)	**18-29 years**	36.1	(33.1-39.3)
Low education	48.5	(42.1-55.0)	Low education	37.0	(30.5-44.0)
Medium education	51.5	(48.3-54.8)	Medium education	34.4	(30.7-38.3)
High education	53.7	(48.7-58.7)	High education	42.1	(36.0-48.4)
**30-44 years**	53.1	(51.1-55.1)	**30-44 years**	39.5	(36.9-42.2)
Low education	42.5	(35.8-49.5)	Low education	37.4	(29.5-46.1)
Medium education	54.5	(51.8-57.2)	Medium education	38.9	(35.4-42.6)
High education	56.2	(53.0-59.3)	High education	41.3	(37.6-45.1)
**45-64 years**	48.7	(46.8-50.6)	**45-64 years**	33.7	(31.9-35.4)
Low education	43.0	(38.6-47.6)	Low education	27.4	(23.3-31.9)
Middle education	48.8	(46.5-51.2)	Middle education	32.3	(29.9-34.7)
High education	53.4	(50.6-56.2)	High education	38.3	(35.8-40.9)
**≥65 years**	42.7	(40.5-44.9)	**≥65 years**	33.5	(31.6-35.6)
Low education	39.7	(36.2-43.3)	Low education	31.5	(27.0-36.4)
Middle education	43.8	(40.5-47.2)	Middle education	32.1	(29.2-35.1)
High education	49.9	(44.3-55.5)	High education	37.5	(34.0-41.1)
**Total (women and men)**	**42.1**	**(41.2-43.0)**	**Total (women and men)**	**42.1**	**(41.2-43.0)**

CI=confidence interval
